# Peri-orbital foreign body: a case report

**DOI:** 10.1186/1752-1947-6-91

**Published:** 2012-03-26

**Authors:** Antonio Moretti, Melissa Laus, Domenico Crescenzi, Adelchi Croce

**Affiliations:** 1 ENT Department, University "G D'Annunzio" of Chieti-Pescara, Hospital "SS Annunziata", Via dei Vestini, I-66100 Chieti, Italy

**Keywords:** peri-orbital foreign body, case report, diagnostic imaging, surgery

## Abstract

**Introduction:**

Foreign bodies inside the orbital cavity are rare. They can cause more or less serious complications, depending on their nature and size.

**Case presentation:**

We report a case of a work-related accident involving a peri-orbital foreign body. The patient was a 50-year-old Caucasian man whose face was injured on the right side while he was working with an agricultural machine. On admission, he was fully conscious and did not have any neurological deficits. He had no loss of vision or ocular motility, but had a laceration of the lateral side of his right upper eyelid. A computed tomographic scan revealed a 6-cm-long bended metal object lodged in the lateral bulbar space of the right orbit. The patient recovered well after surgery and a course of antibiotic therapy.

**Conclusion:**

The original aspects of this case are the singularity of the foreign body and its relative harmlessness in spite of its large size.

## Introduction

Penetrating orbitocranial injuries are quite common in military practice, but they very rarely occur in civilian life, where they are predominantly accidental injuries. They are usually due to a high-velocity injury, such as a gunshot or an industrial accident, but also to relatively trivial trauma [1,2]. Orbital foreign bodies are more commonly observed in men than in women and in younger rather than older people. They may result in severe structural and functional damage to the eye or other orbital contents. The management and prognosis depend on the composition and location of the foreign body as well as the possible presence of secondary infection.

An intra-orbital foreign body is an object that lies within the orbit but outside the ocular globe. These objects can be classified according to their composition as (1) metallic, such as steel; (2) non-metallic, which may be inorganic, such as glass; and (3) organic, such as wood or vegetable matter. In general, injuries caused by metal and glass are well-tolerated and, if they do not have any symptoms or signs, may be left *in situ*, whereas organic matter, such as wood and vegetable matter, is poorly tolerated, triggers an intense inflammatory reaction and needs to be removed urgently [3]. Injuries caused by metallic objects and glass are more frequent than organic foreign bodies, probably because, despite modern imaging methods, they are often difficult to identify and locate.

Surgery is planned on the basis of the size and nature of the foreign body (organic objects are usually poorly tolerated), the location (anterior or posterior orbit) and the presence of other injuries or foreign body-related complications (such as optic nerve compression, infections and extraocular muscle involvement) [4]. Foreign body injuries in the orbital region can be approached with a combination of clinical suspicion, basic knowledge and diagnostic tests. The skill and experience of the surgeon are fundamental to decreasing the risk of iatrogenic injuries [5,6].

## Case presentation

**Figure 1:**
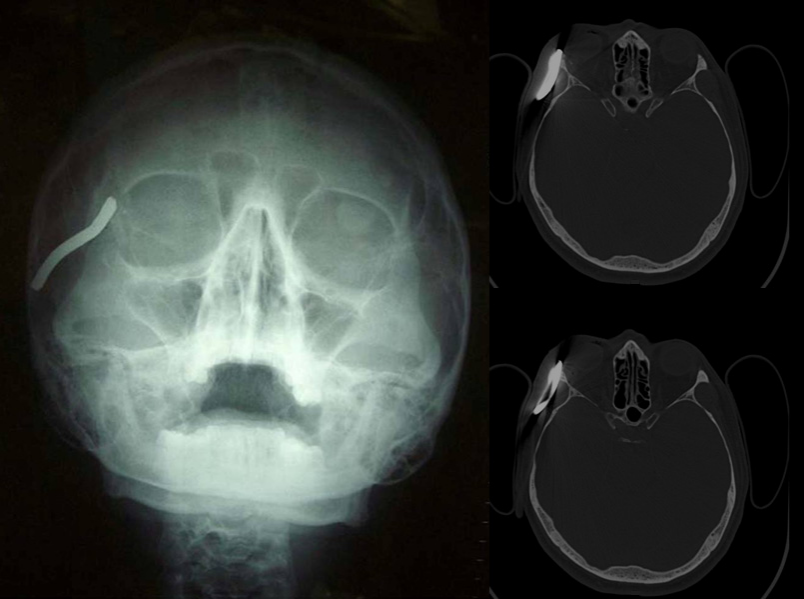
**Cranial X-rays and some axial sections of the computed tomographic scan**.

A 50-year-old Caucasian man who had sustained an injury to the right side of his face while working with an agricultural machine presented to our hospital. He had a completely negative medical history and was not taking any drugs. Cranial X-rays were supplemented by a computed tomographic (CT) scan without contrast enhancement but with multiplanar reconstructions. The CT scan showed a linear metallic foreign body in the soft tissues of the right parietal region (6 cm long, 0.6 cm wide). Anteriorly, this object crossed the lateral wall of the right orbit, determining bone fracture, and small bone and metal fragments and air bubbles could be seen. Posteriorly, the object was localized in the soft tissues over the zygomatic arch. There were no significant alterations in the brain parenchyma. The ventricular system was in place and regular in shape and size (Figure 1).

**Figure 2:**
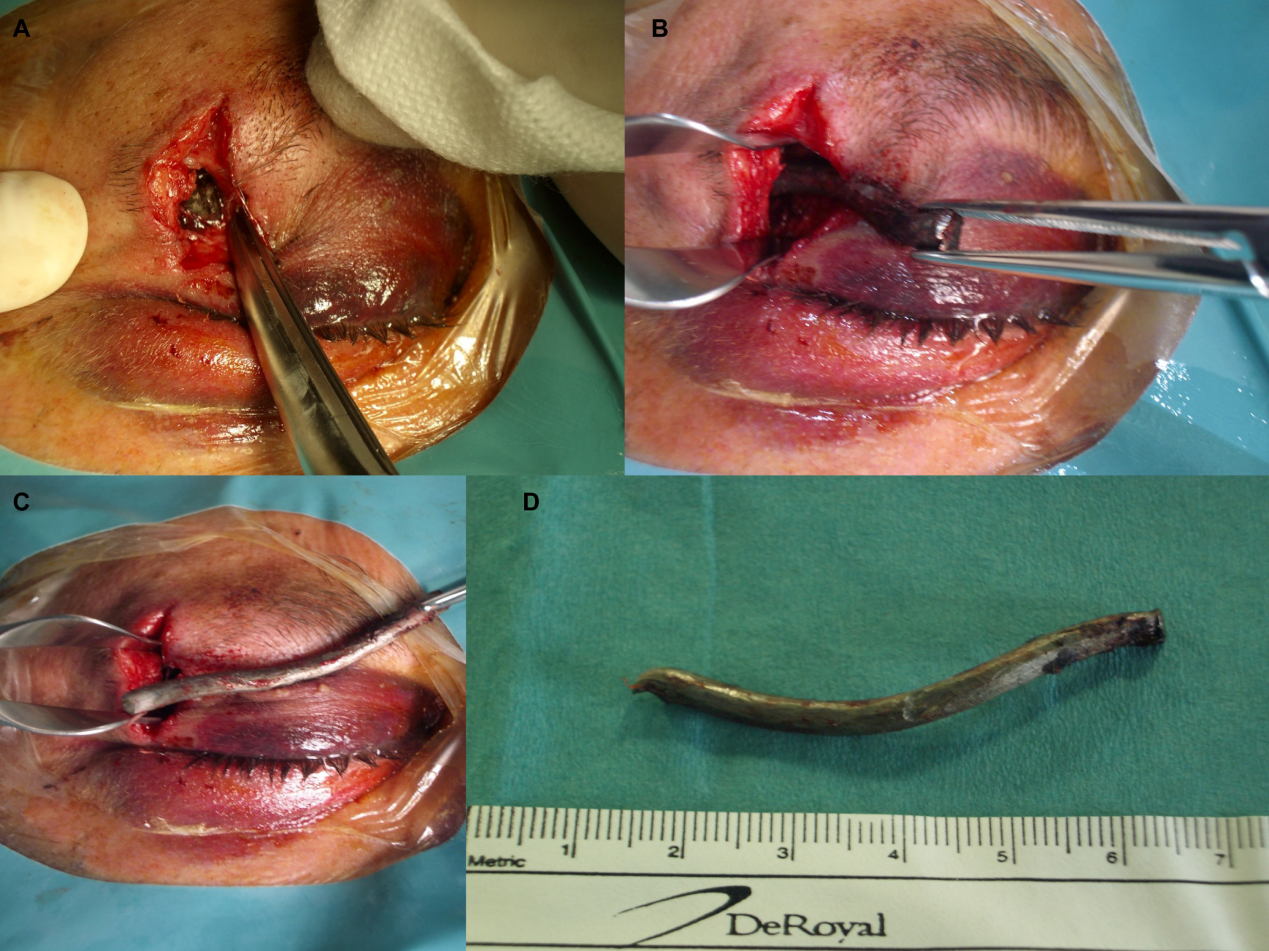
**Intra-operative images of the foreign body**.

**Figure 3:**
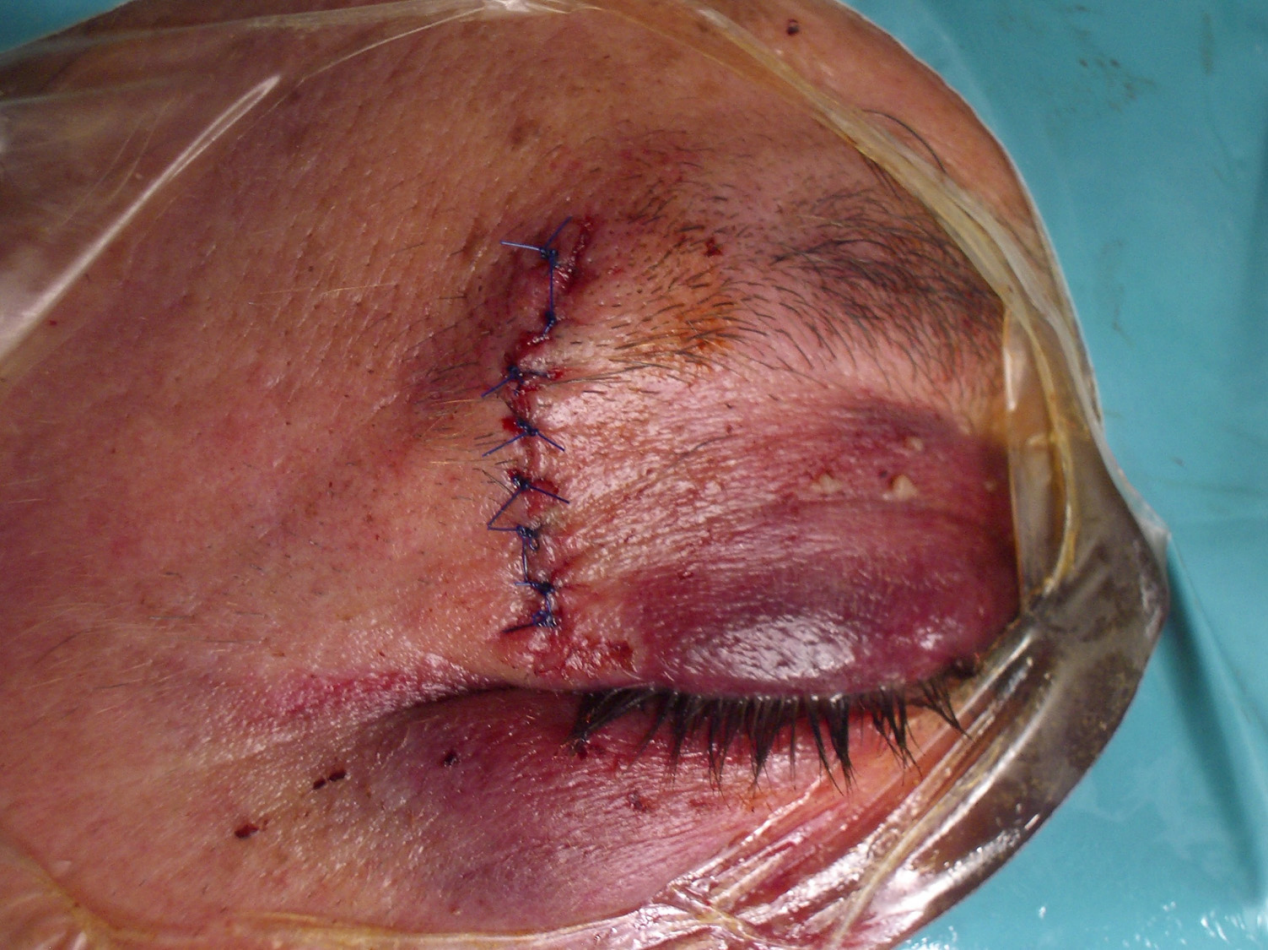
**Patient after surgery**.

The patient's physical examination revealed eyelid hematoma; sub-conjunctival hemorrhage; transparent horny, normal papilla; absent fascicular reflex; and retinal hemorrhages in the superior-temporal septal quadrant. The patient received analgesic medication and prophylactic antibiotics. He then underwent surgery while under general anesthesia. The foreign body was easily extracted through the external injury, located lateral to the outer part of the upper right eyelid. It was metallic, smooth and twisted and measured 6 cm × 0.6 cm (Figure 2). It was extracted from the lateral wall of the orbit, which appeared fractured. We overlapped this fracture with a plate of TUTOPATCH (Med&Care, Gdynia, Poland) for external reinforcement. The injury was sutured in layers and medicated (Figure 3). The patient was discharged with antibiotic therapy, a corticosteroid in decreasing doses and eye drops with the same drugs. One week later, when the patient returned to our department for removal of the stitches, his eyelid bruising had completely resolved.

## Discussion

We report this case for several reasons. First, the patient's condition was unusual because of the type of accident and the proximity of the foreign body to one of the "noble" organs of our body (the eyeball). The clinical examination of patients with an impacted object in the face should be carried out systematically [7]. Active wound bleeding, increasing hematoma, a low hemoglobin level, signs of hypovolemic shock (indications of an associated vascular injury), ocular acuity and mobility (frequently associated with ocular trauma) upon admission should be investigated [8-10].

Our patient was examined by an ophthalmologist, who performed right-eye biomicroscopy (BOD), and examined right-eye tone (TOD), right-eye visual acuity (VOD) and right-eye fundus (FOD). The BOD showed eyelid hematoma, subconjunctival hemorrhage and transparent cornea; the TOD was 18 mm in breadth; the VOD was 7 of 10 natural; and the FOD showed retinal hemorrhages in the upper temporal limits in the papilla and absence of reflection of foveolae. Plain radiography is usually the first additional examination to be requested, owing to its low cost and easy access. It may be useful in identifying and locating intra-orbital foreign bodies, with detection rates of 69% to 90% for metallic foreign bodies and 71% to 77% for glass; however, the detection rate for organic material, such as wood, is low (0% to 15%) [11,12]. If foreign bodies are small and/or of weak density, magnetic resonance imaging MRI should be carried out [13].

The second point of interest is that reverse penetration of the foreign body is very important in terms of surgical removal. The history of an orbital or penetrating eyelid injury should always raise suspicion of an embedded intra-orbital foreign body, particularly in the case of a high-velocity injury. Clinically, the presentation varies, with the patient being asymptomatic or having visual disturbances (decreased vision, double-vision), pain or swelling. The nature of the injury and the foreign object can be ascertained by taking a detailed history. Assessment through radiological images assists in the proper localization of the foreign body, estimation of its consistency and size and evaluation of the response of surrounding orbital tissue. CT scanning has been recommended as the imaging modality of choice in this situation. Despite their being highly sensitive and specific for detection of foreign bodies, however, CT scans may produce false-negative findings, particularly if the size of the foreign body is less than 0.5 mm and especially in the case of wooden objects. In our present case report, the foreign body was located outside the lateral wall of the right orbit and was demarcated clearly by CT scans of the orbit.

Third, priority should be given to the selection of the most logical treatment strategy. The best management of retained metallic intra-orbital foreign bodies remains controversial [14,15]. A retained metallic intra-orbital foreign body may cause a variety of signs, symptoms and clinical findings on the basis of its size, location and composition [14]. Loss of vision is usually due to the initial trauma and is generally not influenced by surgical intervention [15]. Anteriorly located foreign bodies can easily be removed, whereas foreign bodies located more posteriorly without any clinical features should be left where they are, as their removal may result in serious complications [15]. In our patient, because the neurological and radiological investigations showed no vascular injury, we decided to extract the foreign body surgically.

## Conclusion

Management of orbital foreign bodies should include an accurate and detailed history as well as a CT scan of the orbit, which is the imaging modality of choice for detection and localization of the foreign body. The final outcome and prognosis depend greatly upon early diagnosis, followed by surgical exploration and extraction when indicated. Foreign body injuries in the orbital region can be treated with a combination of clinical suspicion, basic knowledge and diagnostic tests and call for surgical skill and experience to decrease the risk of iatrogenic injury in relation to the inherent risk of retaining an organic intra-orbital foreign body.

## Consent

Written informed consent was obtained from the patient for publication of this case report and any accompanying images. A copy of the written consent is available for review by the Editor-in-Chief of this journal.

## Abbreviations

BOD: right eye biomicroscopy; FOD: right eye fundus; TOD: right eye tone; VOD: right eye visual acuity.

## Competing interests

The authors declare that they have no competing interests.

## Authors' contributions

AM and AC were the doctors to whom the patient presented. AM and AC discussed the treatment procedure which followed. AM performed surgery on the patient. AM, AC and DC conducted the patient's post-operative follow-up. All authors contributed to writing the manuscript. All authors read and approved the final manuscript.
